# Treatment of Rheumatic Fever with Salicylic Acid

**Published:** 1876-08

**Authors:** 


The Treatment of Rheumatic Fever with Salicylic Acid.—Like all new remedies, salicylic acid is being tested in a number of different diseases, and among these of course rheumatic fever occupies a prominent place, not only on account of its frequency, but also of its remarkably febrile character. The results which have been obtained in Prof. Traube's wards at Berlin, certainly afford not a little encouragement to indulgence in a hope that in salicylic acid a real remedy for acute rheumatism has at last been found. We extract the following details from a paper by Staff-Surgeon Stricker, in the *Berliner Klinische Wochenscrift* of January 3d, 1876. For several months all the cases of acute rheumatism in which the local symptoms were strongly marked (fourteen in all), were treated with salicylic acid. The preparation used was, however, not the ordinary impure commercial acid, but one that by repeated crystallization had been rendered almost perfectly pure. Thus prepared it consists of shining white needles, which have no smell, and dissolve completely in water and alcohol so as to form a clear solution. This pure acid can be given internally in considerable doses without any of those unpleasant results which have followed the use of the commercial acid, which probably owes its caustic properties to the presence of other substances—for instance, carbolic acid. The pure acid only excites some dryness in the mucous membrane of the mouth and pharynx, followed by an increased secretion from their surfaces. This inconvenience can, however, be obviated by giving the acid in half-gramme or gramme doses every hour, in the form of powder and enclosed in a capsule; and in the treatment of rheumatic fever the administration is continued until the joints which were previously affected can be moved without pain. To quote Dr. Stricker's words, "All the patients thus treated were not only relieved of their fever, but also of the local symptoms—*i. e.*, the swelling, redness, and especially the painfulness of their joints—within forty-eight hours; most of them even within a much shorter period." The largest quantity of pure salicylic acid which was found necessary to produce this effect was fifteen grammes, (230 grains,) and the smallest five grammes; but that even larger quantities can be taken internally, without injuring the digestive apparatus, is proved by the fact that one patient actually took twenty-two grammes in the course of twelve hours, through an excess of zeal on his own part; but, nevertheless, his tongue became clean, and his appetite returned in the course of this vigorous drugging. As far as Dr. Stricker's observations go, the more acute the case the better the action of the acid. He finds it best to begin the treatment in the morning, for then its effects are generally so decided by the evening that it is unnecessary to disturb the patient's rest to give him his medicine. The general phenomena which were observed to follow large doses of the acid were copious perspiration, ringing in the ears, and slight deafness, and in two cases the patients became more than usually lively. Dr. Stricker does not pretend to express any opinion at present on the effect exerted by the acid on the cardiac complications of rheumatic fever. Most of his cases had either old valvular disease or else were suffering from a recent endocarditis at the time of their admission. The details of five cases are appended to Dr. Stricker's paper, and the temperature-sheets are given in graphic form in four of them, and we can only say that they confirm in the most striking manner what has heen above stated as to the value of the salicylic treatment.—*Medical Times and Gazette*, Feb. 5th, 1876, from *Berliner Klin. Wochensch.*

